# Supplementary material for: Comparative multiomics analysis of cell physiological state after culture in a basket bioreactor
Source: Sci Rep. 2022 Nov 23;12:20161. doi: 10.1038/s41598-022-24687-4 (PMC9686226; doi:10.1038/s41598-022-24687-4)
Supplement: Supplementary file 1 — Supplementary Information 1. [file 41598_2022_24687_MOESM1_ESM.zip › raw data/Transcriptomic raw data/7.AS/2.dasplot/TE1vsTC1/RI/9_CEP72_NW_023666064.1_27639108_27639301_+@NW_023666064.1_27639108_27639561_+@NW_023666064.1_27639383_27639561_+.pdf]

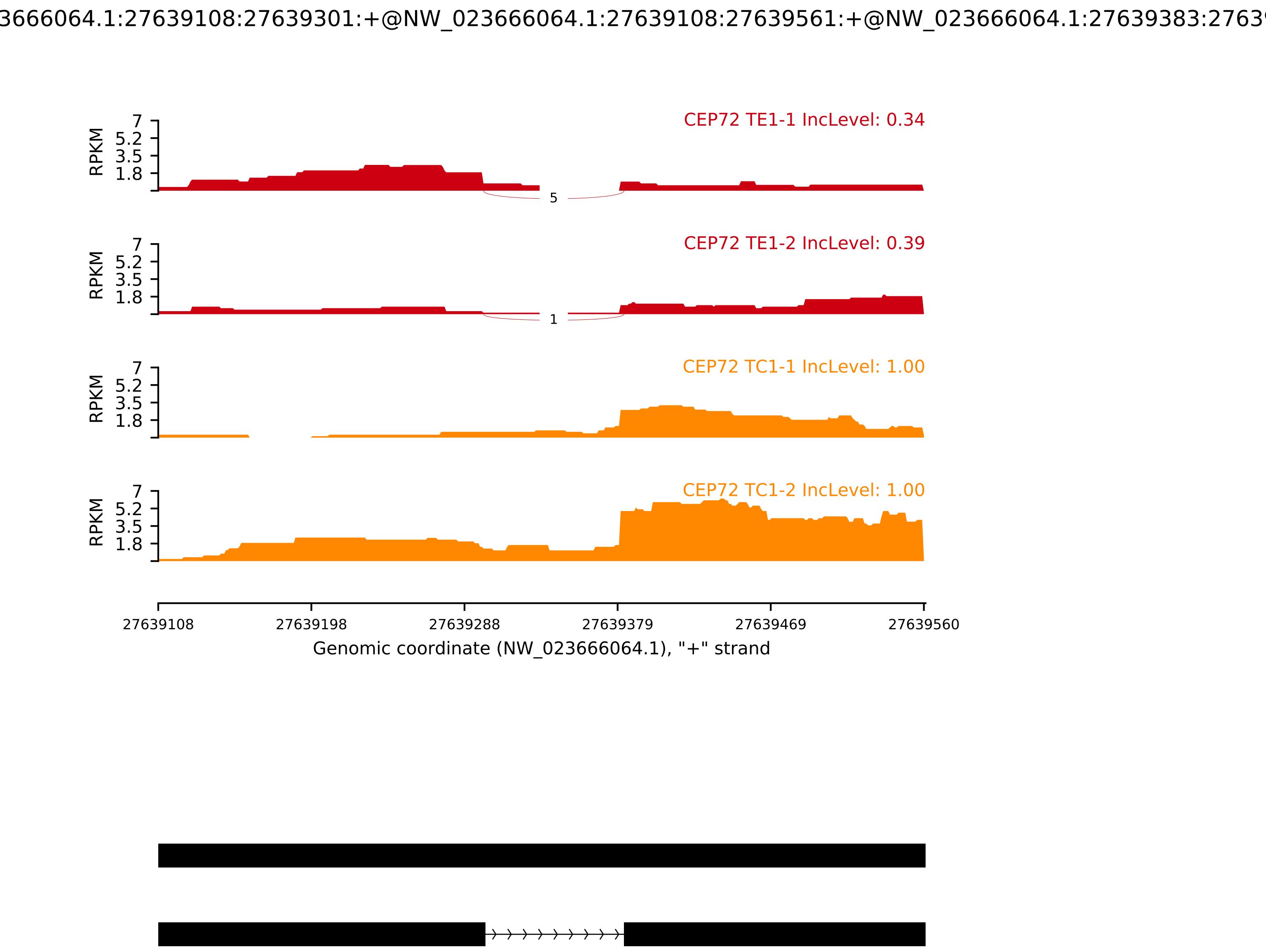

3666064.1:27639108:27639301:+@NW_023666064.1:27639108:27639561:+@NW_023666064.1:27639383:2763
CEP72 TE1-1 IncLevel: 0.34
CEP72 TE1-2 IncLevel: 0.39
CEP72 TC1-1 IncLevel: 1.00
CEP72 TC1-2 IncLevel: 1.00
RPKM
7
5.2
3.5
1.8
5
1
27639108
27639198
27639288
27639379
27639469
27639560
Genomic coordinate (NW_023666064.1), "+" strand
